# Dental and periodontal condition by sociodemographics in Finnish adults in 2023: cross-sectional results from the Healthy Finland Survey

**DOI:** 10.2340/aos.v84.44370

**Published:** 2025-08-19

**Authors:** Suominen Anna Liisa, Leskinen Anni, Saxlin Tuomas, Palotie Ulla, Gursoy Ulvi Kahraman, Sipilä Kirsi, Rautava Jaana, Peltomäki Timo, Lundqvist Annamari, Harjunmaa Ulla

**Affiliations:** a Institute of Dentistry, University of Eastern Finland, Kuopio, Finland; b Odontology Teaching Unit, Kuopio University Hospital, Kuopio, Finland; c Welfare Epidemiology and Monitoring Unit, Finnish National Institute for Health and Welfare, Helsinki, Finland; d Department of Oral and Maxillofacial Diseases, University of Helsinki, Helsinki, Finland; e Oral Diseases Teaching and Dental Care Unit, Helsinki University Central Hospital Head and Neck Center, Helsinki, Finland; f Institute of Dentistry, University of Turku, Turku, Finland; g Research Unit of Population Health, University of Oulu, Oulu, Finland; h Medical Research Center, Oulu, Oulu University Hospital and University of Oulu, Finland; i Department of Pathology, HUSLAB, Helsinki, Finland; j Faculty of Medicine and Health Technology, Tampere University, Tampere, Finland; k Department of Ear and Oral Diseases, Tampere University Hospital, Tampere, Finland; l Welfare Epidemiology and Monitoring Unit, Finnish Institute for Health and Welfare, Helsinki, Finland; m Department of Healthcare and Social Welfare, Unit of Services, Finnish Institute for Health and Welfare, Helsinki, Finland

**Keywords:** Oral health survey, dental caries, periodontal condition, adults

## Abstract

**Objective:**

To obtain current information of adult’s dental and periodontal condition and contributing sociodemographic factors.

**Material and methods:**

Clinical examinations were conducted in 2023 (*n* = 1,798). Enamel and dentine caries prevalences were recorded as the percentages of those having at least one tooth with enamel or dentine caries, and prevalence of periodontitis as those having at least two teeth with clinical attachment loss of ≥ 4 mm or with a probing pocket depth (PPD) of ≥ 6 mm. The numbers of teeth with enamel caries, dentine caries, and PPD (≥ 4 and ≥ 6 mm) indicated the extent of dental and periodontal disease. Sociodemographic factors included age, sex, educational level, and native language.

**Results:**

Two-thirds of the participants had nearly full dentition. Of the dentate participants, 39% had dentine caries and 92% had enamel caries. Periodontitis was detected in 27%, teeth with PPD ≥ 4 mm in 74%, and bleeding on probing in 91% of the dentate participants. Male sex, older age, a lower educational level, and a native language other than Finnish or Swedish were significantly associated with indicators of poorer dental and periodontal conditions in this study – except for extent of enamel caries. The number of teeth affected by enamel caries was highest among younger age groups.

**Conclusion:**

Dental caries and periodontal diseases remain significant health concern among Finnish adults, as do sociodemographic disparities. These findings underscore the importance of implementing targeted preventive interventions for the identified risk groups.

## Introduction

Population-based health examination surveys including clinical examination of oral health are needed not only to assess the prevalence of and trends in oral health problems, but also to enable the identification of risk factors. Self-reported measures from questionnaires and interviews are beneficial, but clinical examinations are needed because the main oral diseases, dental caries, and periodontitis, cannot be reliably self-assessed. Furthermore, registers do not provide comprehensive information on the oral health status of the population, since the information in registers is based on those who visit oral health care and only a proportion of adults regularly use oral health care services.

In Finland, comprehensive clinical health examinations including oral health were conducted in 1978–1980 [1], 2000 [2], and 2011 [3]. Based on the findings of the earlier national surveys, there has been a steady decrease in edentulousness and an increase in the numbers of teeth in the dentate population over the decades. In addition, there has been an improvement in dental health, that is a decreased number of decayed teeth was reported in Finland until the beginning of the 2000s. However, the Health 2000 Survey was the last nationally representative clinical examination. In the earlier Health 2011 Survey, the oral health was examined only in southern and northern Finland. There is therefore a significant need for new and up-to-date information. In addition, the previous national surveys in Finland lack information on novel parameters such as enamel caries or erosive tooth wear.

Information on periodontal health is less conclusive. Taking the number of teeth with deepened periodontal pockets (probing pocket depth [PPD] ≥ 4 mm) as the key indicator of periodontal condition, the periodontal health of the Finnish population has not improved over the decades. One natural explanation for this is the decrease in edentulism. As people retain their teeth for longer, older adults may now have more deepened periodontal pockets than in the past.

In addition to assessing the overall prevalence of and trends in oral health, it is also important to know how oral health is distributed according to demographics and socioeconomics. In Finland, the universal health care system aims to offer unrestricted access to oral health care services for each individual and eliminate disparities in the population. To achieve this, a major reform was implemented in 2001–2002, and since then Finnish citizens have been entitled to public oral health care with subsidised fees or services in private practice, where costs have partly been reimbursed by the National Sickness Insurance. However, persistent sociodemographic and socioeconomic differences in self-reported oral health exist [4]. The initial positive effects of the major oral health reform were seen as a decrease in both the perceived need for oral care and perceived poor oral health. Nevertheless, the small reduction in inequities in self-rated oral health detected in a 3-year follow-up was only temporary, as inequities returned to the previous levels or even widened in a 5-year follow-up [5, 6]. However, from 2000 to 2011, the need for restorative dental treatment decreased simultaneously with a decrease in education-related inequality [7]. Nevertheless, current information on sociodemographic differences or changes in clinically determined dental or periodontal health is lacking in Finland.

This study assessed the dental and periodontal condition of Finnish adults in relation to sociodemographics in 2023 based on a nationwide health examination survey.

## Material and methods

**Figure 1:**
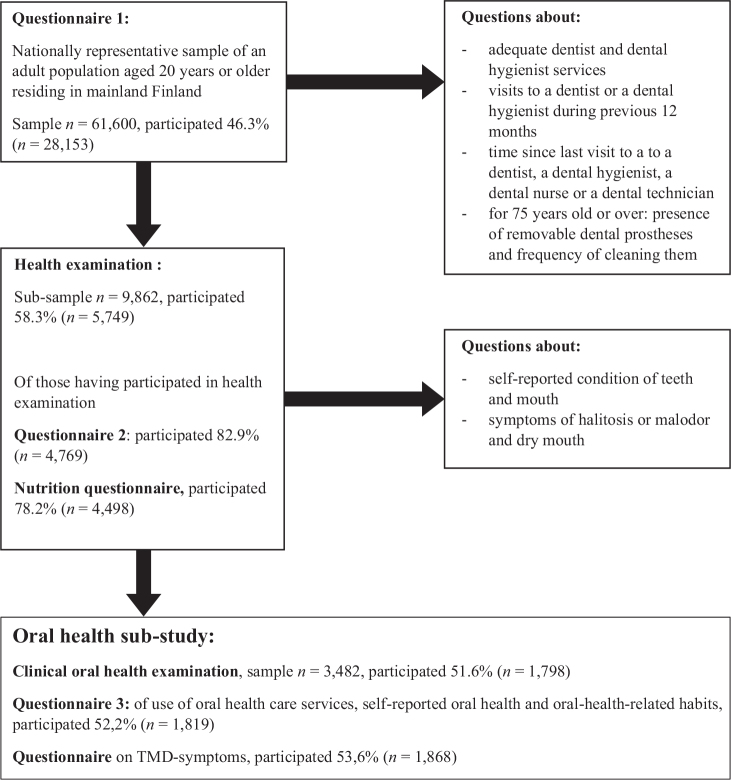
Oral health as part of the Healthy Finland Survey.

The national Healthy Finland Survey was conducted by the National Institute for Health and Welfare in 2022–2023 by using stratified, two-stage cluster sampling, questionnaires, and by carrying out health examinations [8]. Questionnaire 1 of the survey was targeted at a nationally representative sample of the adult population aged 20 years or older residing in mainland Finland (22 counties; *n* = 61,600). The self-administered Questionnaire 1 included questions on health, wellbeing, quality of life, functioning, use of services, lifestyle, and background information. Oral health section included questions regarding the use of oral health care services, as well as self-reported oral health and related symptoms (Figure 1). For the health examination part, a sub-sample of participants (*n* = 9,862) were invited to an extensive health examination conducted between January and June 2023. During the health examination, participants were asked to complete two questionnaires, one on health, living habits and functional ability (Questionnaire 2), and the another on nutrition. The participation rate for Questionnaire 1 was 46.3% (*n* = 28,153) and for the health examination part it was 58.3% (*n* = 5,749). Of those who participated in the health examination, 82.9% (*n* = 4,769) completed Questionnaire 2 and 78.2% (*n* = 4,498) completed the nutrition questionnaire. Further details of the survey are available in Sääksjärvi et al. [8].

### Oral health sub-study

All subjects were invited to participate in at least one additional sub-study, one of which focused on oral health. Participants were informed and recruited at the beginning of the health examination by trained study nurses. Stratified, two-stage cluster sampling was also applied in oral health sub-study. The sample (*n* = 3,522) included the 15 largest cities in mainland Finland, from which 50.0% of the inhabitants were invited to participate. In addition, two health centres from each of five university hospital districts were randomly selected and all of the residents in their catchment areas were invited to participate. Two of the randomised health centres were excluded due to logistic problems. After excluding the deceased and those whose address was not available, the final sample comprised 3,482 people of whom 51.6% (*n* = 1,798) participated in the clinical oral health examination (Figure 1), which was conducted between January and July 2023.

Those who were invited to participate in the clinical oral health examination were requested to complete Questionnaire 3 which included questions on self-reported oral health, oral health-related habits, and the use of services. Of these, 52.2% (*n* = 1,819) returned the Questionnaire 3. All the questionnaires are available via Finnish Institute for Health and Welfare’s (THL) web page (https://thl.fi/en/research-and-development/research-and-projects/healthy-finland-survey/information-for-researchers/questionnaires).

### Clinical oral health examination

The clinical oral health examinations were conducted in the health centres within the sample area or at the university teaching clinics. These examinations followed the protocols of the Health 2000 and 2011 Surveys [9, 10]. Pilot sessions were held twice at the Oral and Dental Centre of the University of Helsinki, Finland, to determine the time needed for an examination and to identify any flaws in the study protocol, forms, and instructions for the examiners. The first pilot session involved 22 examinations, while the second included 4 examinations with voluntary patients. Following these sessions, necessary modifications were made to the protocol, forms, and instructions. Three experienced dentists (UH, UP, and KS in the first and UH and UP in the second pilot session) who were members of the study planning team conducted the pilot sessions. The training for clinical examiners took place in January 2023 at the same clinic as the pilot sessions. The training session began with a 1-day theory session via a virtual meeting platform, led by dentists specialised in cariology and endodontics, periodontology, prosthetic dentistry and stomatognathic physiology, orthodontics, oral pathology, and oral radiology. The following day, clinical training continued at the same clinic as the pilot sessions. Each procedure, except for radiological imaging, was demonstrated by a dentist specialised in the specific field and involved in teaching at a university dental school and the planning of the study protocol. After the demonstrations, the examiners practised the clinical procedures under supervision. Finally, calibration examinations were conducted in which supervisors and study examiners examined the same patients individually and then compared and discussed the results. Training on the use of electronic forms was organised in January 2023 and repeated twice during the spring.

A dentist conducted the examination, while a dental assistant recorded the findings using an electronic examination sheet. The subjects were first asked: ‘Do you have any health condition for which your doctor or dentist has advised you to require antibiotic protection during dental care?’ If the answer was ‘yes’, or if there were any uncertainties, a periodontal examination was not performed.

The examinations were always conducted in the same order, starting with measuring the extent of opening of the mouth and registration of the jaw joints sounds. Masticatory muscles were palpated if the participant reported temporomandibular disorder symptoms. Any removable dentures were recorded, examined for their condition and function, and then removed for the intraoral examination. After palpating the neck and examining the mucosa of the mouth and malocclusions, the patient chair was lowered into a reclining position. The number and location of teeth and the presence of dental plaque were then determined, followed by the recording of spaces in dental arches due to missing teeth. Next, the condition of the teeth, periodontium, and fixed prosthetics were measured and recorded. The instruments used included an oral mirror, a World Health Organization (WHO) periodontal probe with grading on 3.5, 5.5, 8.5, and 11.5 mm, and a 0.5 mm ball ended tip, three-in-one syringe, cotton rolls, and saliva suction if necessary. Fibreoptic transillumination was used to examine particularly the approximal surfaces of teeth that did not have amalgam restorations. The examiner used loupes with a magnification of at least ×2.5.

#### Number of teeth

Tooth identification started from the most posterior upper right tooth and continued along the upper dental arch to the most posterior tooth on the upper left side. The examination continued from the most posterior lower left tooth along the lower dental arch to the most posterior tooth on the lower right side. Thus, the examination order was all teeth present on the upper right side (sector 1) through the upper left (sector 2) and lower left sides (sector 3), and finishing on the lower right side (sector 4).

Each tooth was identified based on its location and morphology. If there was only one premolar or molar in a sector, the premolar was considered as the first premolar if it was located at maximum of 2 mm from the canine, and the molar was considered as the first molar if it was located at a maximum of 2 mm from the second premolar. If there were only three lower incisors in a perfect line, they were considered as teeth 32, 31, and 41.

If there was a deciduous tooth and an equivalent permanent tooth was not present, findings regarding the deciduous tooth were registered as the equivalent permanent tooth. If both the deciduous and permanent tooth were present, findings were only recorded from the permanent tooth. A tooth was registered as a radix if more than half of the vertical crown surfaces were missing, due to caries, fractures, attrition, or therapeutical reasons, for example an abutment tooth for prosthetics. Other findings, such as supernumerary teeth were recorded as free text.

#### Condition of teeth

The condition of a tooth was examined if any part of it was visible or probed with a periodontal probe. Teeth were dried one sextant at a time and cotton rolls were placed in the buccal sulci. The dryness of the lower teeth was also secured with mandibular saliva suction and lingual cottons rolls if necessary. All surfaces of all teeth were examined and after this, the cotton rolls were moistened and removed from the sulci.

Caries was assessed as coronal or root caries, or radix. Caries findings were recorded based on the International Caries Detection and Assessment System (ICDAS) as enamel caries (ICDAS 1–3) or dentine caries (ICDAS 4–6). In the case of deciding between enamel (ICDAS 3) and dentine caries (ICDAS 4), the more severe option was chosen. According to the Finnish Current Care guidelines (Table S1), a value of 4 would necessitate restorative treatment (cavitated surface, decay has penetrated the fissure and undermined the enamel, or the dentine walls of decay feel soft when probing). If the diagnosis of enamel caries (ICDAS 1–2) was uncertain, caries was not registered. Both active and inactive lesions were registered without distinguishing between them.

A tooth was considered in need of repair if it was fractured or dissolved, if a restoration was fractured or lost, if the tooth was otherwise clearly defective, or had a temporary restoration (but no dentine caries). The Current Care Guidelines criteria for ‘repair of restoration’ or ‘replacement of restoration’ (no secondary caries or marginal ditching) were used to assess the need for repair [11]. Teeth with erosion or abrasion defects extending to the dentine were considered in need of repair if contact with the adjacent tooth had been lost, or if the defect was present on over 50% of the surface. A fracture in need of repair had to clearly extend into the dentine. Negative or positive steps of restorations were not considered as in need of repair. Defects from tooth brushing were not considered as in need of repair, if the defect did not clearly extend for more than half of the thickness of the dentine.

Restorations included conventional direct restorations, indirect ceramic restorations, prosthetic crowns, endo-onlays and laminates. Fissure sealants, retentions made for removable prosthetics or other appliances (e.g. clear aligners) were not considered as restorations. A finding was not registered as a restoration if there were any uncertainties.

#### Recordings

Findings for each individual tooth were registered using a two-digit code (Table 1). The first digit represented information about restorations and the second digit represented the diagnosis (caries). For example, a tooth with a composite restoration and initial caries was recorded with a code 12. If the tooth had other diagnostic findings in addition to caries, the tooth was recorded according to the caries findings. If a tooth had been replaced with an implant, it was recorded as missing (code = 99). Additional information concerning implants was written as free text, for example an implant fixture with a healing abutment.

**Table 1:** Codes indicating condition of teeth in the Healthy Finland Survey in 2023.

Restoration codes	Caries codes
0 No restoration/crown 1 Composite restoration 2 Amalgam restoration 3 Amalgam + composite/glass ionomer 4 Full ceramic onlay 5 Other (glass ionomer, gold, etc.) 6 Prosthetic crown (metal ceramic, ceramic, zirconia, gold) 7 Temporary restoration (IRM^a^ or other) 8 Other (e.g. attachment for Maryland/fibre bridges) 9 Cannot be examined or tooth is missing	0 Intact tooth 1 Intact restoration, no caries 2 Initial caries (ICDAS^b^ 1–3) 3 Reparable, no caries 4 Need of repair and initial caries (ICDAS^b^ 1–3) 5 Crown caries (= dentine caries, ICDAS^b^ 4–6), can also have initial caries (enamel caries ICDAS^b^ 1–3) or need for repair 6 Root caries (active or inactive) 7 Both coronal and root caries (2 separate lesions) 8 Radix, with or without caries 9 Cannot be examined (for example tooth is missing, covered in plaque/calculus or tooth is missing)

ICDAS: International Caries Detection and Assessment System.

a Intermediate Restorative Material;

b International Caries Detection and Assessment System.

The indicators describing dental condition in this study included the prevalences of enamel and dentine caries, which were recorded as the percentages of those having at least one tooth with enamel or dentine caries. The numbers of sound teeth, teeth with enamel or dentine caries, teeth in need of repair with and without dentine caries, and teeth with restorations indicated the extent of dental disease. These indicators were categorised as presented in Table 3.

#### Periodontal condition

The periodontal condition of each tooth, except for third molars and radices (coronal part of the tooth missing with no prosthetic replacement), was assessed by using indices for PPD, clinical attachment loss (CAL) and the presence of bleeding on probing (BOP). Probing pocket depths were measured using a WHO periodontal probe with a probing force of 20 grams on four sites of the teeth (distal angle, midpoint of buccal site, midpoint of lingual site, and mesial angle). The deepest PPD measurement on each tooth was registered as no periodontal pocket, a periodontal pocket of 4–5 mm, or a periodontal pocket of ≥ 6 mm. Clinical attachment loss was determined at the site of the most advanced gingival recession on each tooth. Given that the cemento-enamel junction was clearly distinguishable, the distance from the cemento-enamel junction to the base of the periodontal pocket (gingival recession and PPD in total) was measured to represent CAL, and was registered as follows: no gingival recession, CAL ≤ 3 mm, CAL = 4–5 mm, or CAL ≥ 6 mm. The presence of BOP was assessed and registered as ‘no’ or ‘yes’ for each tooth after the measurements of PPD and CAL. If the periodontal parameters could not be assessed, they were registered as ‘cannot be measured’.

The indicators of periodontal condition used were the occurrence of periodontitis and the numbers of teeth with deepened (PPD ≥ 4 mm) and severely deepened (PPD ≥ 6 mm) periodontal pockets, as well as the proportion of those having BOP in ≥10% of teeth. A periodontitis case was defined as a participant having at least two teeth with CAL ≥ 4 mm or with PPD ≥ 6 mm.

### Sociodemographics and covariates

Sociodemographic characteristics included in this study were age, sex, educational level, and native language. The age of participant was determined in years and categorised in the analyses into categories of 20–34, 35–44, 45–54, 55–64, 65–74, and ≥ 75-years. The educational level was retrieved from Statistics Finland (https://stat.fi/en/luokitukset/koulutusaste/koulutusaste_1_20160101). The national Classification of Education 2016 is primarily meant for national statistical reporting. It is used for classifying education obtained by the population, the labour force, employees and other such personnel groups and individual people, as well as for the education activities of educational institutions. The classification is based on UNESCO’s International Standard Classification of Education 2011 (https://uis.unesco.org/sites/default/files/documents/isced-2011-en.pdf). It was initially categorised into early childhood education, primary education, lower secondary education, upper secondary education, post-secondary non-tertiary education, short-cycle tertiary education, bachelor’s or equivalent level, master’s or equivalent level, doctoral or equivalent level, and not elsewhere classified. The educational level was further categorised into three categories: lower (= early childhood education, primary education, lower secondary education), middle (= upper secondary education, post-secondary non-tertiary education), or higher (= short-cycle tertiary education, bachelor’s or equivalent level, master’s or equivalent level, doctoral or equivalent level). The native language was categorised into Finnish, Swedish, or other. Other covariates included the area of residence (five collaborative areas for healthcare and social welfare) and the number of teeth.

### Statistical analyses

To obtain representative results for the target population, the Healthy Finland survey used stratified cluster sampling. The data analyses were performed using SAS Callable SUDAAN software (Release 11.0) to take into account the two-stage cluster sampling design and by using weight coefficients to also account for the non-response rate. Weighting coefficients in the analyses were calibrated with the original strata so that different sampling probabilities were taken into account. For the analyses, two university hospital districts (1 and 5) were merged into a new stratum 6, so that each stratum had at least two clusters. Weights were produced using the Inverse Probability Weighting method and the following data available for the entire sample were used for the calculation: age, sex, marital status, socioeconomic group, occupation, native language, the urbanity of the area of residence, and time since the previous hospitalisation (separately for periods of cardiovascular disease, mental health problems, infections, births, and accidents).

Comparisons between sexes and according to sociodemographics were based on descriptive statistics, such as distributions, mean and median values, standard errors of the means, and interquartile ranges. Statistical evaluation was carried out by means of chi-squared tests and regression analyses. Poisson regression models with robust error variance [12] were used to examine the associations between sociodemographics and the occurrence of dentine caries and periodontitis, as well as the number of teeth with enamel or dentine caries and the number of teeth with deepened or severely deepened periodontal pockets. The regression analyses were adjusted for the area of residence and the number of teeth.

### Ethics approval

For the questionnaire part of the survey, the ethical review was conducted by the institutional review board (IRB) of THL. Completing the questionnaire was considered to indicate consent. For the health examination part of the survey, the survey was reviewed by the Helsinki and Uusimaa Hospital District Regional Committee on Medical Research Ethics (decision number HUS/900/2022). Written informed consent was obtained from each individual participating in the health examination. Written feedback from the oral examination specified whether treatment was needed and, if so, what kind of treatment was recommended.

## Results

Of the complete sample, those who participated in the clinical oral health examination were older, more highly educated, and more often women and native Finnish speakers than those who did not participate (Table 2).

**Table 2:** Distribution of study participants and non-participants in the clinical oral health examination: The Healthy Finland Survey in 2023.

Sociodemographic and -economic background and morbidity	Clinical oral health examination		*p* ^a^
	Participated *n* = 1,798	Did not participate *n* = 1,684	
	%	
**Age group (years)**			< 0.001
20–34	20	31	
35–44	17	17	
45–54	15	14	
55–64	19	14	
65–74	18	10	
75+	11	14	
**Women**	56	49	< 0.001
**Level of education**			< 0.001
Lower	13	26	
Middle	40	46	
Higher	47	28	
**Native language**			< 0.001
Finnish	91	88	
Swedish	3	2	
Other	6	10	
**Occupation**			< 0.001
Managers	2	1	
Professionals	15	10	
Technicians and associate professionals	13	9	
Clerical support workers	4	3	
Service and trades workers	10	11	
Skilled agricultural, forestry and fishery workers	1	1	
Craft and related trades workers	4	6	
Plant and machine operators, and assemblers	3	4	
Elementary occupations	3	4	
Unknown	45	51	
**Socioeconomic group**			< 0.001
Student	6	7	
Manual worker	12	17	
Lower-level employees with administrative and clerical occupations	23	19	
Upper-level employees with administrative, managerial, professional and related occupations	17	12	
Self-employed person	5	4	
Pensioner	28	26	
Others	9	15	
**Marital status**			< 0.001
Married	49	35	
Unmarried	32	40	
Divorced or separated	13	14	
Widow	5	7	
Unknown	1	4	
**Statistical grouping of municipalities**			0.064
Urban	79.7	77.7	
Rural	0.1	0.4	
Semi-urban	20.2	21.9	
**Urban-rural classification**			0.305
Inner urban area	50	49	
Outer urban area	22	19	
Peri-urban area	11	13	
Local centres in rural areas	11	12	
Rural areas close to urban areas	3	3	
Rural heartland areas	3	4	
Sparsely populated rural areas	1	1	
**Hospitalisation from care Notification System (HILMO)**			
Any ICD-10 code	96	94	0.001
Cardiovascular disease (ICD-10 codes beginning with I)	30	26	0.017
Mental health (ICD-codes beginning with F)	17	27	< 0.001
Infectious diseases (ICD-10 codes beginning with A, B or J0–J2)	30	33	0.119
Birth (ICD-codes beginning with O)	21	18	0.035
Accidents (ICD-codes beginning with S, T, V, X or Y)	48	48	0.930

a chi square test between the participants and non-participants.

**Table 3:** Weighted^a^ findings for dentate participants except for number of teeth. Statistical evaluation by Chi squared tests.

Clinical findings	Men	Women	All
All	*n* = 785	*n* = 992	*n* = 1,777
	% (95%CI^b^)		
**No. of teeth**			
0	3 (1–9)	2 (1–5)	3 (1–6)
1–19	9 (7–11)	9	9 (7–11)
20–24	12 (10–15)	14 (12–16)	13 (11–15)
25–32	75 (71–79)	75 (70–79)	75 (71–79)
	*p* = 0.388		
**Dentate**			
Condition of teeth	*n* = 772	*n* = 977	*n* = 1,749
**No. of sound teeth** (no restorations, need of repair, dentine or enamel caries)			
0	5 (3–7)	4 (3–6)	4 (3–6)
1–4	12 (8–17)	13 (10–17)	12 (9–16)
5–14	37 (30–46)	34 (30–39)	36 (30–42)
15+	46 (36–57)	49 (41–57)	47 (39–56)
	*p* = 0.172		
**No. of teeth with enamel caries** (ICDASt^c^ 1–3)			
0	6 (4 –0)	9 (7–11)	8 (6–10)
1–4	22 (16–30)	25 (21–29)	24 (20–28)
5–10	38 (33–44)	39 (35–43)	38 (33–44)
11+	33 (29–38)	28 (23–33)	30 (26–35)
	*p* = 0.025		
**No. of teeth with dentine caries (**ICDAS^c^ 4–6)			
0	55 (47–63)	67 (58–76)	61 (53–69)
1–2	28 (24–32)	22 (19–27)	25 (23–27)
3+	17 (9–29)	10 (6–17)	14 (8–23)
	*p* < 0.001		
**No. of teeth in need of repair without dentine caries**			
0	69 (64–73)	76 (72–80)	72 (69–75)
1–2	27 (23–31)	21 (19–24)	24 (22–26)
3+	5 (4–6)	3 (1–6)	4 (3–5)
	*p* = 0.060		
**No. of teeth with dentine caries or need of repair**			
0	41 (33–50)	55 (49–61)	48 (42–55)
1–2	33 (31–36)	30 (27–33)	32 (30–33)
3+	25 (17–36)	15 (11–21)	20 (14–28)
	*p* < 0.001		
**No. of teeth with restorations**			
0	6 (4–8)	7 (6–9)	6 (5–8)
1–4	20 (16–26)	20 (18–21)	20 (18–23)
5–14	31 (27–35)	28 (25–32)	30 (27–33)
15+	43 (34–52)	45 (42–48)	44 (39–49)
	*p* = 0.257		
Periodontal condition	*n* = 741	*n* = 937	*n* = 1,678
**No. of teeth^d^ with deepened (PPD^e^ ≥ 4 mm) periodontal pockets**			
0	19 (13–27)	32 (24–42)	26 (19–34)
1–2	20 (17–23)	23 (19–28)	22 (18–25)
3–7	28 (22–34)	27 (23–32)	27 (24–32)
8+	33 (26–41)	17 (11–26)	25 (19–33)
	*p* < 0.001		
**No. of teeth^d^ with pockets (PPD^d^ ≥ 6 mm) deepened periodontal pockets**			
0	74 (69–79)	86 (80–90)	81 (76–85)
1–2	15 (11–19)	10 (7–14)	12 (10–16)
3–7	8 (6–10)	3 (1–6)	5 (4–8)
8+	3 (2–6)	1 (< 1–2)	2 (1–3)
	*p* < 0.001		
**Prevalence of periodontitis** ^f^			
	*n* = 742	*n* = 934	*n* = 1,676
	34 (28–41)	20 (15–26)	27 (22–33)
	*p* < 0.001		
**Proportion of those having bleeding on probing ≥ 10% of teeth**	*n* = 831	*n* = 937	*n* =1,679
	95 (91–97)	88 (85–90)	91 (89–92)
	*p* < 0.001		

a Based on age, sex, marital status, socioeconomic group, occupation, native language, urbanity of the area of residence, and time from previous hospitalisation (separately from periods of cardiovascular disease, mental health problems, infections, births and accidents).

b 95% Confidence Interval

c International Caries Detection and Assessment System

d Excluding third molars and radices.

e Probing pocket depth.

f A periodontitis case was defined as participant having at least two teeth with either clinical attachment loss ≥ 4 mm or with probing pocket depth ≥ 6 mm

While 3% of the participants had lost all their teeth, 75% had almost full dentition, men as often as women (Table 3). Edentulous participants were only found in the older age groups (8% of the participants aged 65–74 years and 13% of those aged ≥ 75 years). Enamel caries was detected in 92% of the participants and dentine caries in 39%, and half of the participants (52%) had either dentine caries or teeth in need of another type of repair. Two thirds (74%) of the participants had teeth with deepened (PPD ≥ 4 mm) periodontal pockets and 19% had teeth with severely deepened (PPD ≥ 6 mm) periodontal pockets whereas periodontitis was observed in 27% of the participants. Almost all (91%) of the participants had BOP in ≥10% of teeth.

### Occurrence of dentine caries and periodontitis by sociodemographics

The occurrence of teeth with dentine caries and periodontitis were more common findings in men than in women, and highest in older age groups (Table 4). Similar trends were observed among participants with a lower educational level and those who were not native Finnish or Swedish speakers.

**Table 4:** Weighted^a^ prevalences (%) of any teeth with dentine caries and periodontitis among the dentate participants by sociodemographics

Sociodemographic factors	Any teeth with dentine caries (ICDAS^b^ 4–6) *n* = 1,749	Periodontitis^c^ *n* = 1,676
	% (95%CI^d^)	
**All**	39 (31–47)	27 (22–33)
**Sex**		
Men	45 (37–53)	34 (28–41)
Women	33 (24–42)	20 (15–26)
	*p* < 0.001	*p* < 0.001
**Age group (years)**		
20–34	37 (31–43)	6 (4–10)
35–44	37 (24–52)	19 (13–28)
45–54	31 (20–45)	24 (20–29)
55–64	41 (31 51)	31 (25–38)
65–74	37 (27–48)	50 (38–68)
75+	54 (40–67)	52 (41–62)
	*p* < 0.001	*p* < 0.001
**Level of education**		
Lower	43 (34–53)	29 (24–35)
Middle	35 (28–42)	23 (17–30)
Higher	33 (27–40)	25 (18–32)
	*p* = 0.009	*p* = 0.033
**Native language**		
Finnish	38 (30–47)	26 (21–33)
Swedish	28 (15–46)	25 (15–38)
Other	53 (42–63)	33 (20–48)
	*p* = 0.032	*p* = 0.614

a Based on age, sex, marital status, socioeconomic group, occupation, native language, urbanity of the area of residence, and time from previous hospitalisation (separately from periods of cardiovascular disease, mental health problems, infections, births and accidents).

b International Caries Detection and Assessment System

c A periodontitis case was defined as participant having at least two teeth with either clinical attachment loss ≥ 4 mm or with probing pocket depth ≥ 6 mm

d 95% Confidence Interval

In the adjusted regression analyses (Table 5), men, participants with a lower educational level, and participants who were not native Finnish or Swedish speakers had a higher probability of having both dentine caries and periodontitis compared to women, those with a higher level of education, and native Finnish speakers. While the probability of periodontitis was higher in older than in younger age groups, this trend was not that clear for dentine caries.

**Table 5:** Adjusted^a^ associations of sociodemographics with occurrence of dental caries and periodontitis among the dentate participants by Poisson regressions with robust variance.

Sociodemographic factors	Any teeth with dentine caries (ICDAS^b^ 4–6) *n* = 1,607	Periodontitis^c^ *n* = 1,540
	PR (95% CI^d^)	
**Sex**	*p* < 0.001	*p* < 0.001
Men	**1.4 (1.2**–**1.6)**	**1.9 (1.6**–**2.2)**
Women	ref.	ref.
**Age group (years)**	*p* < 0.001	*p* < 0.001
20–34	ref.	ref.
35–44	1.1 (0.7–1.8)	**3.3 (2.0**–**5.3)**
45–54	0.9 (0.5–1.5)	**4.2 (2.4**–**7.4)**
55–64	1.2 (0.9–1.8)	**5.9 (3.4–10.2)**
65–74	1.1 (0.8–1.5)	**8.8 (5.4**–**14.3)**
75+	**1.5 (1.0–2.1)**	**9.0 (5.4**–**15.2)**
**Level of education**	*p* = 0.002	*p* = 0.025
Lower	**1.3 (1.1**–1.5**)**	**1.2 (1.0**–**1.5)**
Middle	1.1 (0.9–1.2)	1.0 (0.9–1.2)
Higher	ref.	ref.
**Native language**	*p* < 0.001	*p* = 0.041
Finnish	ref.	ref.
Swedish	0.7 (0.5–1.1)	1.2 (0.8–1,8)
Other	**1.5 (1.2**–**1.8)**	**2.2 (1.2**–**4.0)**

a Adjusted for the area of residence and the number of teeth.

b International Caries Detection and Assessment System.

c A periodontitis case was defined as participant having at least two teeth with either clinical attachment loss ≥ 4 mm or with probing pocket depth ≥ 6 mm

d Prevalence Ratio,with 95% Confidence Interval

### Extent of caries and periodontal disease by sociodemographics

**Figure 2:**
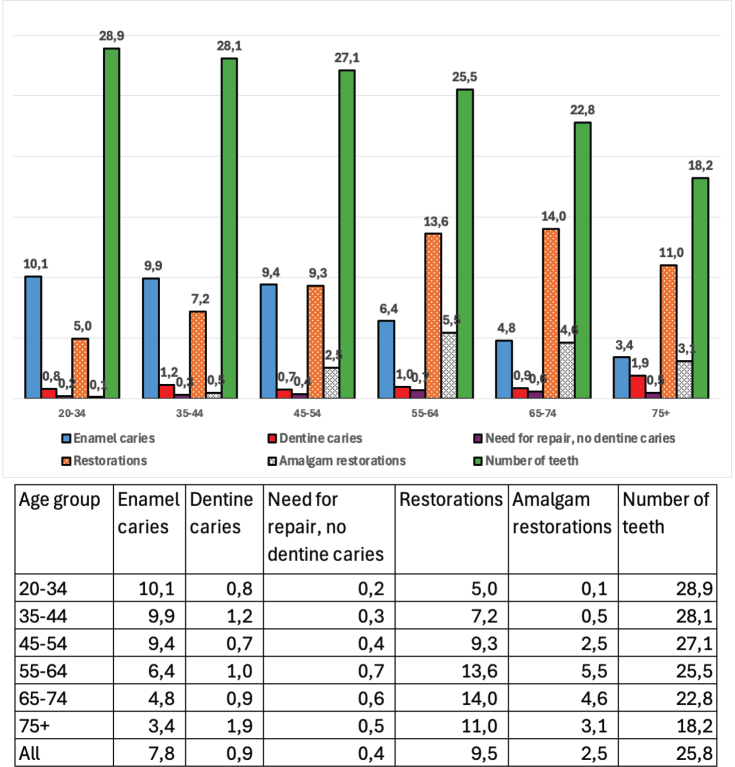
Mean numbers of teeth and teeth with enamel caries, dentine caries, need for repair (no dentine caries), restorations, and amalgam restorations by age group for dentate participants.

Men and those who were not native Finnish or Swedish speakers had higher mean number of teeth with enamel or dentine caries and teeth with deepened and severely deepened periodontal pockets than women or native Finnish speakers (Table 6). The mean number of teeth with enamel caries was highest in the youngest age group whereas the mean number of teeth with dentine caries and with restorations was highest in the oldest age groups (Figure 2). The highest mean number of teeth with deepened periodontal pockets was seen among the middle-aged. Those having the highest educational level had the highest number of teeth with enamel caries but the lowest number of teeth with dentine caries. This group also had a lower number of teeth with deepened periodontal pockets than the lower educated participants.

**Table 6:** Dental and periodontal findings in terms of weighted^a^ medians, interquartile ranges (IQR), means and standard errors (SE) for dentate participants.

Sociodemographic factors	No. of teeth with enamel caries (ICDAS^b^ 1–3)	No. of teeth with dentine caries (ICDAS^b^ 4–6)	No. of teeth^c^ with deepened (PPD^d^ ≥ 4 mm) periodontal pockets	No. of teeth^c^ with severely deepened (PPD^d^ ≥ 6 mm) periodontal pockets
	Median (IQR)/Mean (SE)		Median (IQR)/Mean (SE)	
	*n* = 1,749		*n* = 1,678	
**All**	6.9 (7.9)/7.8 (0.3)	0 (0.8)/1.0 (0.2)	2.3 (7.0)/4.8 (0.5)	0 (0)/0.7 (0.1)
**Sex**				
Men	7.2 (8.0)/8.1 (0.4)	0 (1.1)/1.3 (0.4)	3.5 (8.7)/6.0 (0.6)	0 (0.1)/1.0 (0.2)
Women	6.6 (7.8)/7.5 (0.3)	0 (0.5)/0.8 (0.1)	1.5 (5.2)/3.8 (0.5)	0 (0)/0.4 (0.1)
**Age group (years)**				
20–34	9.8 (7.9)/10.1 (0.5)	0 (0.6)/0.8 (0.1)	1.1 (4.6)/3.5 (0.3)	0 (0)/0.1 (0.04)
35–44	9.3 (6.6)/9.9 (0.4)	0 (0.6)/1.2 (0.6)	2.8 (9.6)/5.7 (0.5)	0 (0)/0.7 (0.2)
45–54	8.6 (7.2)/9.4 (0.6)	0 (0.4)/0.7 (0.2)	3.8 (8.3)/6.1 (1.0)	0 (0)/0.9 (0.3)
55–64	5.4 (5.6)/6.4 (0.3)	0 (1.0)/1.0 (0.2)	2.9 (7.2)/5.4 (0.8)	0 (0.1)/1.0 (0.3)
65–74	3.3 (5.6)/4.8 (0.3)	0 (0.8)/0.9 (0.2)	1.8 (5.8)/4.3 (0.7)	0 (0.1)/0.7 (0.1)
75+	1.9 (4.5)/3.4 (0.3)	0.2 (2.3)/1.9 (0.5)	2.5 (5.9)/4.1 (0.5)	0 (0.3)/0.8 (0.1)
**Level of education**				
Lower	7.0 (7.8)/7.8 (0.4)	0 (1.0)/1.1 (0.3)	2.8 (7.7)/5.2 (0.5)	0 (0)/0.9 (0.1)
Middle	6.8 (8.3)/7.7 (0.4)	0 (0.6)/1.0 (0.3)	1.9 (6.5)/4.6 (0.6)	0 (0)/0.4 (0.1)
Higher	7.1 (7.9)/8.0 (0.3)	0 (0.5)/0.8 (0.1)	1.8 (5.5)/3.9 (0.3)	0 (0)/0.4 (0.1)
**Native language**				
Finnish	6.8 (7.9)/7.6 (0.3)	0 (0.8)/1.0 (0.2)	2.2 (6.7)/4.7 (0.6)	0 (0)/0.6 (0.1)
Swedish	6.2 (6.6)/6.8 (0.8)	0 (0.2)/0.7 (0.2)	0.8 (5.7)/4.2 (1.0)	0 (0)/0.4 (0.2)
Other	9.2 (9.5)/10.0 (0.6)	0.1 (1.4)/2.1 (0.8)	5.1 (10.3)/6.9 (0.6)	0 (0.2) 0.9 (0.3)

a Based on age, sex, marital status, socioeconomic group, occupation, native language, urbanity of the area of residence, and time from previous hospitalisation (separately from periods of cardiovascular disease, mental health problems, infections, births and accidents).

b International Caries Detection and Assessment System.

c Excluding third molars and radices.

d Probing pocket depth.

In the adjusted regression analyses (Table 7), men and those who were not native Finnish speakers had a higher probability of having a greater number of teeth with dentine caries and teeth with deepened (PPD ≥ 4 mm) periodontal pockets than women or native Finnish speakers. Those belonging to the older age groups and having a lower educational level had higher probabilities of both a higher number of teeth with dentine caries and teeth with deepened and severely deepened periodontal pockets than those in younger age groups or having higher level of education. Those belonging to the oldest age groups had lower probabilities of having a higher number of teeth with enamel caries than participants in younger age groups.

**Table 7:** Adjusted^a^ associations of sociodemographics with number of teeth with initial or dentinal caries or deepened periodontal pockets among the dentate participants by Poisson regressions with robust variance (RR: Rate Ratio; CI: Confidence Intervals).

	No. of teeth with enamel caries (ICDAS^b^ 1–3)	No. of teeth with dentine caries ICDAS^b^ 4–6)	No. of teeth^c^ with deepened (PPD^d^ ≥ 4 mm) periodontal pockets	No. of teeth^c^ with severely deepened (PPD^d^ ≥ 6 mm) periodontal pockets
	RR (95% CI)			
	*n* = 1,607		*n* = 1,542	
**Sex**	*p* = 0.267	*p* < 0.001	*p* < 0.001	*p* = 0.013
**Men**	1.0 (1.09–1.1)	**1.7 (1.4**–2.0**)**	**1.5 (1.3**–**1.7)**	2.2 (1.2–4.3)
**Women**	ref.	ref.	ref.	ref.
**Age group (years)**	*p* < 0.001	*p* < 0.001	*p* < 0.001	*p* < 0.001
**20–34**	ref.	ref.	ref.	ref.
**35–44**	1.0 (0.9–1.1)	1.7 (0.6–4.8)	**1.6 (1.4**–1.9**)**	**6.1 (3.0**–**12.3)**
**45–54**	1.0 (0.9–1.1)	1.1 (0.6–2.1)	**1.9 (1.3**–**2.7)**	**7.8 (1.9**–**31.9)**
**55–64**	**0.7 (0.7**–**0.8)**	1.7 (1.0–2.94	**1.9 (1.6**–**2.3)**	**8.8 (4.2**–**18.5)**
**65–74**	**0.6 (0.5**–**0.7)**	1.4 (0.8–2.5)	**1.9 (1.5**–**2.4)**	**9.1 (5.0**–**16.6)**
**75+**	**0.5 (0.4**–**0.6)**	**2.8 (1.3**–6.3**)**	**2.1 (1.7**–**2.7)**	**12.5 (6.1**–**25.5)**
**Level of education**	*p* = 0.945	*p* = 0.038	*p* < 0.001	*p* < 0.001
** Lower**	1.0 (0.8–1.2)	**1.4 (1.1**–1.9**)**	**1.4 (1.2**–**1.6)**	**2.7 (1.7**–**4.0)**
** Middle**	1.0 (0.9–1.1)	**1.4 (1.1**–**1.9)**	1.2 (1.09–1.5)	1.2 (0.7–2.2)
** Higher**	ref.	ref.	ref.	ref.
**Native language**	*p* = 0.861	*p* < 0.001	*p* < 0.001	*p* = 0.017
** Finnish**	ref.	ref.	ref.	ref.
** Swedish**	0.9 (0.8–1.2)	0.8 (0.5–1.3)	1.09 (0.7–1.4)	1.3 (0.4–3.5)
** Other**	1.0 (0.9–1.1)	**2.4 (1.6**–**3.8)**	**1.9 (1.5**–**2.3)**	2.6 (1.3–5.1)

a Adjusted for the area of residence, and the number of teeth.

b International Caries Detection and Assessment System

c Excluding third molars and radices.

d Probing pocket depth.

## Discussion

Almost all the adults in this study were dentate, and two-thirds of the participants had nearly full dentition. Dentine caries was detected in two-fifths of the participants. Periodontitis was detected in one-third of the examined individuals, but teeth with deepened periodontal pockets (PPD ≥ 4 mm) were found in two-thirds. Sociodemographic differences were prominent, with male sex, older age, a lower educational level, and a native language other than Finnish or Swedish being associated with a poorer dental and periodontal conditions in terms of both occurrence and extent. Enamel caries was detected almost in all participants with the highest prevalence and extent found among younger age groups.

### Edentulism and tooth loss

According to our findings, the previously observed trend [2, 3] of decreasing edentulism and increasing tooth counts in Finland had continued. The differences between sexes had already levelled off earlier. Similar results from national clinical examinations have been reported globally: in 2015 [13], in the USA between 2017 and 2020 [14], in Norway between 2017 and 2019 [15], in Germany in 2014 [16], and earlier (around the 2010s) in the UK [17], Hungary [18], and South-Korea [19]. A recent questionnaire-based study in the UK in 2021 similarly demonstrated that 95% of adults in England were dentate, with an average of 25.4 natural teeth among dentate adults [20].

### Dentine caries

In the present study, 39% of the examined adults had dentine caries. This is slightly higher than the 31% reported in the Health 2000 Survey [2] and significantly higher than the 23% reported in 2011 [3]. The primary reason for these differences is most likely the differences in the caries recording method. In this Healthy Finland Survey, the ICDAS classification was used, with a lower threshold for recording dentine caries. In the Health 2000 and 2011 Surveys, registered dentine caries corresponded to ICDAS scores of 5–6, whereas in the present study, ICDAS scores of 4–6 were recorded as dentine caries. This approach was chosen because ICDAS scores of 4–6 typically correspond to caries lesions that require restorations in both public dental services and private practice in Finland. In addition, examiners in the present study used loupes, which was not the case in the previous surveys. Another reason could simply be the increased number of teeth, leading to a higher risk of oral diseases. Moreover, the data in the Health 2011 Survey were only collected from southern and northern parts of Finland. Adults in southern Finland have had the best oral health in Finland, while those in northern Finland have had the lowest number of teeth [2]. This probably led to an underestimated prevalence of dentine caries in 2011.

The prevalence observed in this study is, however, similar to that reported in the Global Burden of Disease 2015 study [13], in which caries in permanent teeth occurred in 34% of the participants. Furthermore, the prevalence of caries was 31% among adults over 16 years old in the UK in 2009 [21], 32% among adults over 15 years old in Australia in 2017–2018 [22], and 33% among adults over 20 years old in Sweden in 2013 [23]. In contrast, in Norway 56% of the examined individuals between 2017 and 2019 had dentine caries [15], and this difference was explained by varying recording methods and more rural target population. However, in the US between 2005 and 2008, just over one in five (22%) adults aged >20 years had untreated dental caries [24], with almost no differences between age groups. Lower figures were also seen in Korea [25] where the overall prevalence of caries among those aged ≥15 years was 26%, with the highest prevalence (32%) in the younger (aged 20–29 years) and the lowest prevalence (24%) in the oldest (aged 70–79 years) age groups. In this study, the highest prevalence of caries occurred in the oldest age groups, being 37% among those aged 65–74 years and 54% among those aged ≥75 years. Regarding older age, this is in line with a review by Kassebaum et al. [26], which suggests that the burden of untreated caries is shifting from children to adults, with three peaks in prevalence at the ages of 6, 25, and 70 years. Similarly, in Germany, the increase in the number of decayed teeth was more pronounced among older adults (aged 65–74 years) than among younger adults (aged 35–44 years) between 1997 and 2014 [16]. This shift was also reported in Sweden [23].

### Periodontal pocketing

In terms of the number of teeth with deepened periodontal pockets, the periodontal condition of Finnish adults has not improved during recent decades. In the Health 2000 Survey, 64% of adults aged 30 years or older had teeth with deepened periodontal pockets, whereas 21% had teeth with severely deepened (PPD ≥ 6 mm) periodontal pockets [2]. Eleven years later, the Health 2011 Survey [3] found that 64% of the participants still had teeth with deepened periodontal pockets, while 18% had severely deepened periodontal pockets. Moreover, the distributions of the number of teeth with deepened periodontal pockets according to sex and age followed similar patterns as in the previous Finnish national surveys.

### Periodontitis

In this study, a little less than one-third (27%) of participants had periodontitis, with men more often affected than women and older participants more often than younger ones. According to the Global Burden of Disease 2019 study, the relative increase in the number of prevalent cases of periodontitis in Finland from 1990 to 2019 was modest (0–50%), and is at the same level as in other European countries and the US [27]. It was estimated that the age-standardised prevalence (ASR) rates of periodontitis in 2019 in Finland [28] were on the same level as in Norway, higher than in Sweden, and lower than in Denmark. Regarding the estimated ASR rates and other European countries, Germany was at the same level, while others fared better than Finland [28]. In the US, in 2009–2014 [29], an estimated 42% of dentate adults aged 30 years or older had periodontitis, with 8% having severe periodontitis which is consistent with global reports in 2019 [28]. According to the 2018 Korea National Health and Nutrition Examination Survey (KNHANES) VII [30], the prevalence of periodontal disease in Korea was significantly lower (23%).

### Bleeding on probing

Bleeding on probing was common in this study, reflecting either transient gingival inflammation or established gingivitis or periodontitis, indicating that only one in 10 had a healthy periodontium. Similarly, in the UK in 2009 [17], only 17% of dentate adults had completely healthy periodontal tissues and no periodontal disease (that is no bleeding, no calculus, no periodontal pocketing of ≥4 mm, and in the case of adults aged 55 or above, no loss of periodontal attachment of ≥4 mm anywhere in any tooth). In Hungary, in 2003 [31], healthy periodontal conditions, defined as no BOP (Community Periodontal Index (CPI) = 0), were only found in 12%, while shallow periodontal pockets (PPD 4–5 mm) were observed in 23%, and deep pockets (PPD ≥ 6 mm) in 7% of the examined population.

In general, it appears that periodontal health has not improved over the last two or three decades in Finland, Europe, or outside Europe, including the US. Instead, the global burden of the disease has increased substantially since 1990 [27], with the highest increase observed in South Asia and East Asia and in 2019, the largest ASR-prevalence was observed in Sub-Saharan Africa. The Global Burden of Disease 2018 study [28] suggests three reasons for this; population growth, population aging, and changes in age-specific prevalence rates.

### Dental and periodontal condition by sociodemographics

Sociodemographics refers to the social and demographic characteristics of a specific group of individuals, such as age, sex, educational level, and income. In our study, poorer dental and periodontal condition was found among men, those with a lower educational level, and who were not native Finnish or Swedish speakers. In Finland, Finnish and Swedish are the official languages, while native speakers of other languages are typically immigrants. In 2023, 85% of Finnish citizens were speakers of Finnish, 5% Swedish, and 10% had other languages as their native language. These findings were confirmed by adjusted regression analyses, indicating their independent effects. This suggests that the sociodemographic gradient has persisted in Finland despite efforts to address the unequal availability and use of services and the resulting health disparities. Socioeconomic differences favouring the highly educated remained unchanged.

Similar findings have been reported in other Nordic countries, for instance in Sweden [23], Denmark [32] and Norway [33], which have very similar health care organisations, as well as in other European countries such as in Hungary [31], and outside Europe in Australia [22] and globally [27]. Enamel caries was an exception as it particularly affected younger individuals, a pattern also observed in Norway [17]. Regarding enamel caries, there were no significant differences in relation to other sociodemographic factors examined in our study. Another Finnish study based on the North Finland Birth Cohort (NFBC 1966) also found that the frequency of enamel caries lesions (ICDAS scores 1–3) did not significantly differ between sexes, but their study only included 46-year-olds [34]. However, in that study, enamel caries was prevalent in almost the entire study population (99%), and lesions needing restorative treatment (ICDAS 4–6) were prevalent in 40%, similarly to our study. It is natural for dentine caries to be more common in older individuals and enamel caries in younger individuals. Although examining enamel caries in population-based surveys is a relatively novel practice, it is important, because it indicates the risk of developing dental caries and aids in the planning of targeted prevention. As suggested by Skeie et al. [35], the potential for preventing the development of more severe caries lesions should be fully exploited. Thus, enamel caries should be a part of epidemiological reporting in national registers. In Finland, as in other Nordic countries, oral healthcare system aims at providing equal access to care. Nevertheless, certain population groups, particularly those in weaker social positions, do not fully benefit from the current healthcare systems. Social factors such as low income, a low educational level, and an immigrant background are still associated with poorer oral health across all Nordic countries [36]. According to Andersen [37], the use of services depends on the individual and living conditions, and never solely on organisations or costs, but on a complex network of societal and social determinants. This may explain why the health disparities in Nordic countries, which are based on the welfare state model, are so persistent and roughly similar to those in other parts of Western Europe [38].

## Strengths and limitations

A strength of this study is the nationally representative sample with comprehensive and multiple measures of dental and periodontal health. This study is part of rich data that allows the possibility to combine information with general health and health behaviour. The well-planned framework and a tested protocol, developed for the previous Finnish surveys in 1980 and 2000 are also strengths of this survey. The measurements in this study ensured comparability with earlier data but also includes some new measurements such as CAL. The current protocol was also carefully tested, and any noted shortages were corrected. A limitation is the low participation rate in the health examination, with the lowest participation rates being among the youngest men. Those who did not attend the survey are probably those with the poorest oral health and the results may thus be too optimistic. However, in international comparison, these participation rates can be considered as good. Nevertheless, background information on the dropouts was available and was used in defining the weights to correct the population-level estimates and non-response. According to Härkänen et al. [39], statistical methods based on weighting or multiple imputation provide quite accurate results when comparing the survey results with data obtained from national registers. Another limitation is the lack of reliability measurements. For logistical reasons, it was not possible to conduct inter- or intra-rater measurements due to the involvement of several examiners and examination locations around the country. However, all the examiners were experienced general practitioners who were trained to work as clinical examiners, particularly in this study. The methods used to ensure the reliability of the examinations included calibration lectures, clinical training with simulated oral health examinations, web-based written instructions, and ongoing consulting possibilities during the examinations. It has also previously been reported that clinical examinations by general practitioners and calibrated examiners are comparable and both are reliable for scientific purposes [40].

An improvement in the periodontal examination of this survey was the measurement of CAL, which allowed more precise estimation of the occurrence of periodontitis than in previous Finnish surveys. However, this means that no comparison can be made with previous surveys of the occurrence of periodontitis. Principles suggested by Papapanou et al. [41] and Holtfreter et al. [42] were applied in the definition of periodontitis case in this study. The case definition (a participant having at least two teeth with CAL ≥ 4 mm or PPD ≥ 6 mm) is a modification of the 2018 AAP (American Academy of Periodontology)/EFP (European Federation of Periodontology) definition [41] and follows a similar approach very recently presented by Tay et al. [34]. However, there are a few exceptions. Firstly, since no site-specific information on CAL was available due to the strict time limitation of the oral health examination in this survey, no distinction could be made between whether CAL was present on interproximal or buccal/oral sites. Therefore, the ‘no-adjacent teeth criterion’ was not adopted. Instead, a CAL threshold of ≥ 4 mm was applied due to the use of the WHO periodontal probe and to avoid overestimation of the occurrence of periodontitis related to the inability to distinguish between interproximal or buccal/oral sites. Secondly, since CAL was only measured at the site of the most advanced gingival recession (given that the cemento-enamel junction was visible), PPD was applied for those teeth with no CAL measurements (no gingival recession or the cemento-enamel junction not visible). Since the PPD thresholds were ≥ 4 mm or ≥ 6 mm (due to the use of the WHO periodontal probe), the threshold of PPD ≥ 6 mm was eventually implemented to avoid overestimation of the occurrence of periodontitis related to the possibility of ‘pseudopockets’ with PPD threshold of ≥ 4 mm and no certainty of CAL being present.

Since the use of periodontal probes with a fine graduation, such as those with single millimetre markings (e.g. the UNC-15 probe), is recommended for epidemiological surveys, the use of the WHO periodontal probe (markings at 3.5, 5.5, 8.5, and 11.5 mm) in this survey can be considered a limitation. Due to this, somewhat crude thresholds of PPD (≥ 4 and ≥ 6 mm) and CAL (≤ 3, ≥ 4, and ≥ 6 mm) were implemented. Yet another limitation is related to the definition of periodontitis in this study (participant having at least two teeth with CAL ≥ 4 mm or PPD ≥ 6 mm). Since CAL was only measured if there was gingival recession (and the cemento-enamel junction was clearly visible), with no distinction made between interproximal and buccal/oral sites, and the thresholds of CAL ≥ 4 mm and PPD ≥ 6 mm were used, the occurrence of periodontitis may have been somewhat underestimated.

Nationwide representative clinical studies in adult populations are scarce because they are expensive and laborious to conduct. Moreover, detailed comparisons between and within populations are complicated because of differences in samples and age groups used for reporting, but especially in clinical measurement methods and in the definitions of indices describing periodontal health. However, comparisons are valuable and an attempt should at least be made.

## Conclusions

Dental caries and periodontal diseases among adults remain significant public health issues in Finland, as do sociodemographic disparities. The increased need for treatment of older age groups is evident in the high number of missing teeth and teeth with dentine caries, as well as the poorer periodontal condition. Moreover, attention should be directed also towards younger age groups where enamel caries is particularly common. This supports a shift in focus from treatment of caries to prevention. Taken together, these findings emphasise the importance of ensuring access to care and implementing targeted preventive interventions for the identified risk groups.
